# Clinical Barriers to Hands-Free, Eyes-Free Voice Input for Nursing Records: Field Usability Study

**DOI:** 10.2196/71462

**Published:** 2026-06-02

**Authors:** Natsuko Nishida, Chang Liu, Goshiro Yamamoto, Tomohiro Kuroda

**Affiliations:** 1 Department of Medical Informatics, Course of Social Informatics, Graduate School of Informatics, Kyoto University, 54 Shogoin-kawahara-cho, Sakyo-ku, Kyoto, 606-8507, Japan, 81 75-366-7701; 2 Department of Nursing, Kyoto University Hospital, Kyoto, Japan; 3 Department of Real World Data R&D, Graduate School of Medicine, Kyoto University, Kyoto, Japan; 4 Preemptive Medicine and Lifestyle Related Disease Research Center, Kyoto University Hospital, Kyoto, Japan; 5 Division of Medical Information Technology and Administration Planning, Kyoto University Hospital, Kyoto, Japan

**Keywords:** feasibility study, workflow, catheterization laboratories, point-of-care documentation, mobile phone

## Abstract

**Background:**

Nursing records are essential for maintaining patient care quality but impose a substantial workload on nurses, thus contributing to burnout and diverting attention from direct care. Voice input technology enables hands-free and eyes-free documentation, allowing simultaneous patient care and record entry. Despite its potential, its adoption in clinical nursing practice remains limited owing to concerns about patient privacy, technical instability, and the complexity of entering structured data into electronic medical record interfaces. Furthermore, most previous studies have been conducted in simulation settings or have focused on post hoc dictation. Thus, the feasibility of true simultaneous documentation in real-world clinical environments remains largely unexplored.

**Objective:**

This pilot study was designed to explore the feasibility of using hands-free and eyes-free voice input for concurrent nursing documentation in the highly structured clinical environment of a catheterization laboratory.

**Methods:**

This study was conducted at Kyoto University Hospital using a mixed methods exploratory design. Eight cases of percutaneous transhepatic cholangiodrainage and transcatheter arterial chemoembolization were observed between December 2022 and January 2023. Five nurses participated in this study and documented intraoperative events using both traditional handwritten records and a prototype voice dialogue system comprising a smartphone (Google Pixel 6a) and wireless earphones (Pixel Buds Pro). Researchers observed the nurses’ behavior in adjacent control rooms to minimize interference. Log data from voice input and the corresponding handwritten notes were compared to determine the proportion of events successfully captured. In addition, semistructured interviews and usability surveys were conducted to obtain qualitative feedback on usability, practicality, and perceived barriers.

**Results:**

Voice input successfully recorded 40% to 100% of events during the preoperative and intraoperative phases, but only 0% to 12% during postoperative documentation. Observations and interviews revealed that the postoperative phase involved higher cognitive and communication demands, thereby making simultaneous voice documentation difficult. A significant barrier identified was the “social awkwardness” of interacting with the system; nurses reported feeling embarrassed when speaking loudly to activate the device and often stepped away from patients to record data, negating the benefit of concurrent entry. Concerns about disturbing patients or interrupting medical communication have also hindered use.

**Conclusions:**

This pilot study identified two major barriers in applying concurrent voice input in clinical settings: (1) the instability and unreliability of using voice input as the sole recording method and (2) the conflict between voice interaction and the social dynamics of care. To overcome these, future implementations should consider visible devices to signal recording status, support for whisper recognition, and protocols for code words to handle sensitive information. Gradual implementation may reduce nurses’ cognitive and psychological burdens. Further large-scale longitudinal studies are warranted to validate these strategies in routine nursing documentation.

## Introduction

Nursing records are not only essential documents of patients’ conditions but also serve as key materials for evaluating the quality of care and ensuring patient safety [1,2]. Nurses and other health care professionals rely on nursing records to understand patient status and coordinate care. Therefore, nurses must update these records promptly and accurately. However, documentation accounts for a substantial proportion of nursing tasks—over 30% of the total working time in some reports [3-5]—and has been identified as a factor contributing to nurse fatigue and burnout [6-8]. Thus, reducing the documentation burden while maintaining accuracy and completeness has become a central issue in nursing practice and informatics research [9-12].

Voice input technology, which enables documentation through speech, has attracted attention as a possible solution to this challenge. If nurses could record information verbally while providing care, they would no longer need to allocate separate time for documentation after completing clinical tasks, potentially reducing total work time. In the future, task-oriented voice dialogue systems customized for nursing scenarios, integrating natural language processing and conversational artificial intelligence, may further support nurses by enabling hands-free and eyes-free sequential documentation. Such systems could allow nurses to make a query on the required data elements, receive prompts regarding missing information, and complete documentation through voice interactions in real time.

Voice input also holds promise for improving the documentation continuity in multitasking environments. Nursing work inherently involves frequent interruptions such as responding to patient calls, communicating with physicians, and handling sudden changes in patient conditions [13]. During such events, nurses often postpone documentation and must rely on memory until they find the time to record it. As record accuracy depends on memory, delayed entry increases the risk of omission or error [14,15]. From this perspective, the ability to document immediately and directly by voice, even when one’s hands are occupied or while maintaining visual attention to the patient, offers an important potential advantage.

However, implementing voice input in clinical settings faces practical and ethical challenges, most notably ensuring a secure input environment that protects patient privacy [16]. Nurses are constantly on the move [3,17]; requiring them to relocate to a private area before dictating would reduce efficiency and negate the benefits of real-time documentation.

Previous studies that have applied speech-to-text technologies to nursing records have primarily used simulated patients or laboratory-based scenarios. Most of these studies have examined voice input for patient care documentation [18-21]. The study by Lee et al [22] reported its use for triage nurse documentation in emergency departments. However, evaluations in actual clinical settings remain limited. As speech recognition accuracy improves, studies have reported improvements in documentation accuracy [18,19], input speed [18,19,22], and nurses’ acceptance and satisfaction [18,19]. Nevertheless, several challenges persist, including the incompatibility of voice input with the complex hierarchical structure of electronic medical records [19], potential interference with nurse-patient communication [20], and the need for training and associated costs [23].

As most of these studies were conducted under simulated conditions, privacy protection measures were not sufficiently addressed. Moreover, these studies generally assumed that nurses would perform voice input while viewing an electronic record interface, thereby overlooking the central benefit of hands-free and eyes-free documentation conducted concurrently with clinical tasks. To date, the feasibility of such real-time voice input in actual clinical settings has rarely been examined, highlighting the need for preliminary field-based investigations.

For hands-free and eye-free documentation to be applied in nursing practice, the clinical setting and type of records must be carefully selected. Situations involving highly variable information, such as patient complaints or subtle physical changes, are unsuitable for this approach. The conditions required for testing voice input include environments in which patient privacy can be adequately protected, and tasks follow standardized procedures with relatively predictable documentation content. In such cases, nurses are not required to deliberate over what to record, as the system is activated only when documentation is needed and returns to standby mode afterward. Moreover, as nurses frequently communicate with both patients and physicians, the system must distinguish between documented utterances and conversational speech.

Therefore, we selected nursing documentation from a catheterization laboratory as the focus of this pilot study. This environment satisfies several key conditions necessary for testing concurrent voice inputs. First, because only 1 patient was present in the room at a time, the major challenge of maintaining patient privacy during voice recordings was mitigated. Second, nursing activities in the catheterization laboratory follow a relatively standardized sequence: patient entry, time-out, procedure, completion, and exit, allowing nurses to document procedural progress, patient responses, and nursing interventions in chronological order. In this context, the variation in record content is smaller than in inpatient wards, where nurses document diverse and frequently changing care situations. Nurses can simply verbalize their observations and actions without the need to view or verify the input on the display. Furthermore, communication between patients and physicians is limited to essential exchanges, which minimize interference with the recording process.

Unlike simulation-based studies, which cannot fully reproduce real clinical tension, urgency, or workload, an in situ approach enables the observation of how voice input interacts with authentic clinical dynamics. Conducting investigations under carefully controlled conditions in actual clinical settings, while minimizing the burden on both patients and nurses, can yield valuable preliminary insights into practical barriers to implementation.

Therefore, this pilot study was designed to explore the feasibility of using hands-free and eyes-free voice inputs for concurrent nursing documentation within the highly structured clinical environment of a catheterization laboratory. Specifically, it aimed to examine (1) how nurses can use voice input during clinical tasks, (2) what contextual and technical barriers might arise, and (3) what conditions are necessary to facilitate integration into practice. As clinical contexts differ across settings, the findings are not intended to be generalized; rather, this preliminary study seeks to identify implementation challenges and provide foundational evidence to guide future large-scale investigations.

## Methods

### Overview of Nursing Operations

This pilot study was conducted in the catheterization laboratory at Kyoto University Hospital, Japan, where two procedures were selected as the study context: percutaneous transhepatic cholangiodrainage (PTCD) and transcatheter arterial chemoembolization (TACE).

These procedures were performed under local anesthesia by inserting an angiographic catheter through the femoral access. PTCD aims to drain bile, whereas TACE involves the administration of anticancer drugs and embolization of the hepatic artery. Both surgeries share several nursing procedures common to other interventional treatments and involve relatively simple, standardized documentation tasks. As these procedures are short and exhibit fewer fluctuations in patients’ vital signs than cardiac or neurological angiographic interventions, they are considered appropriate for minimizing patient risk while allowing real-world investigation under routine conditions.

The catheterization laboratory environment typically maintains a noise level of 60 dB to 75 dB generated by fluoroscopy equipment, computer fans, air conditioning systems, and conversations among health care professionals. Each surgical procedure was performed by several physicians, radiologists, and a nurse. Nurses document observations, interventions, and vital data in short phrases and numerical entries, rather than lengthy descriptive texts. These records were written on a dedicated angiography nursing record sheet and kept in a binder that nurses carried during procedures to enable quick access and recording.

The surgical process is divided into three distinct phases: (1) preoperative phase: from preparation and patient entry to the start of the procedure, (2) intraoperative phase: from time-out to the end of the procedure, and (3) postoperative phase: from completion of the procedure until the patient exits the room.

**Figure 1.**
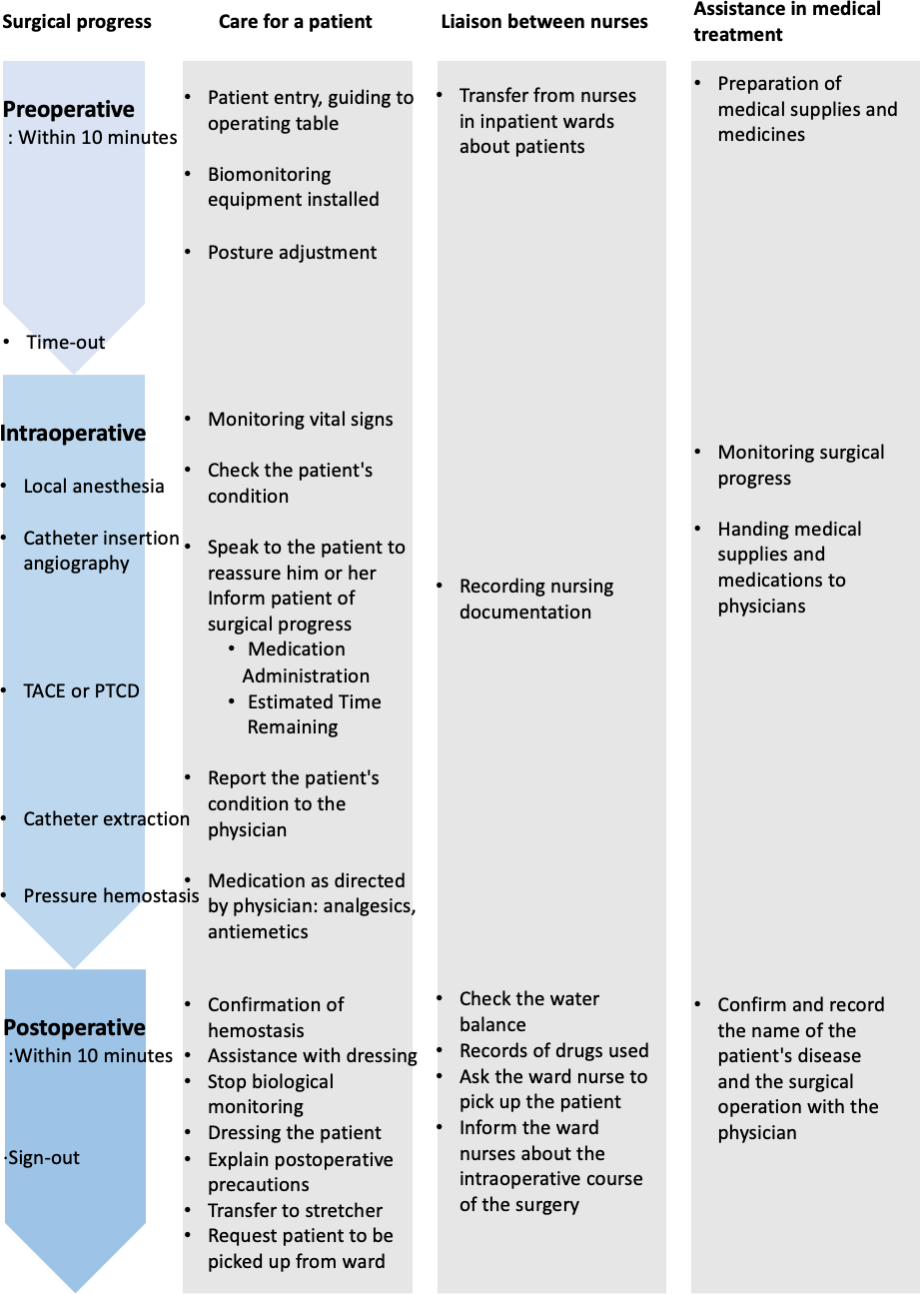
Nurses’ tasks in an angiography room. PTCD: percutaneous transhepatic cholangiodrainage; TACE: transcatheter arterial chemoembolization.

Nurses’ responsibilities during these phases can be broadly categorized into three domains: (1) direct patient care, (2) collaboration with ward nurses and documentation, and (3) assisting physicians, including instrument handling and medication administration. During the preoperative and postoperative phases, numerous tasks overlap within a short time (often less than 10 minutes), creating highly intensive work periods. Figure 1 summarizes the surgical progress and corresponding nursing tasks in each phase.

The nursing documentation in the catheterization laboratory included vital signs, procedural progress, fluid balance, puncture sites and indwelling devices, administration of analgesics and antiemetics, and postoperative instructions. Given the structured nature of these entries, the environment was deemed suitable for examining the feasibility of hands-free and eyes-free voice input without introducing additional patient risks.

### Study Design and Participants

This study adopted a mixed methods exploratory design to identify practical barriers and contextual factors influencing the use of voice input in clinical documentation. This study combined quantitative log data analysis with qualitative observations and interviews.

To ensure patient safety and minimize workflow disruption, the number of cases was limited. On the basis of feasibility and workload considerations, a sample size of ≥5 nurses was established as adequate for this pilot phase. Participants were nurses who had worked in the catheterization laboratory for at least 1 year and were familiar with both PTCD and TACE procedures. Only scheduled procedures were included; emergency cases were excluded to prevent patient risk and ensure stable observation conditions.

During the procedure, the nurses performed routine handwritten documentation while simultaneously using a voice input system to record equivalent information whenever possible. As handwritten records serve as official medical documentation, nurses were explicitly instructed to prioritize them over voice inputs to ensure completeness and patient safety.

The equipment setup was carefully designed to integrate the voice input device into the clinical workflow without interrupting surgical progress. All procedures were observed by the research team, who recorded the operational patterns, timing, and contextual conditions that affected the voice input performance.

### System Setup

This study was conducted within a closed and secure hospital electronic medical record network in accordance with the institutional privacy and data protection policies. All devices used were dedicated exclusively to the study and operated only within this protected infrastructure. No patient-identifiable data were stored outside the hospital system or transmitted externally, thereby eliminating the risk of patient data leakage.

For this pilot study, we used Google Assistant, a voice-activated dialogue application with built-in note-taking functionality [24]. A Google Pixel 6a smartphone with Google Assistant preinstalled and offering stable voice control was used as the main recording device. A Pixel Buds Pro wireless earphone-microphone was selected owing to its lightweight design, comfort, and compatibility with radiation-protection goggles. Although earphones support ambient sound pass-through, only 1 earbud was used to ensure that the surrounding sounds could still be heard and to avoid any potential patient safety risks.

When the nurse said “OK Google,” Google Assistant was activated, followed by “Take a note,” prompting the response “What would you like to note?” The nurse then verbalized the desired record—for example, “The patient has entered the room.” Google Assistant replied, “The note has been saved,” completing the entry within approximately 10 to 15 seconds. The spoken data were immediately transcribed and stored as text in Google Keep. Although the application can also be launched manually from a smartphone, only voice activation was used in this study to maintain a hands-free operation.

**Figure 2.**
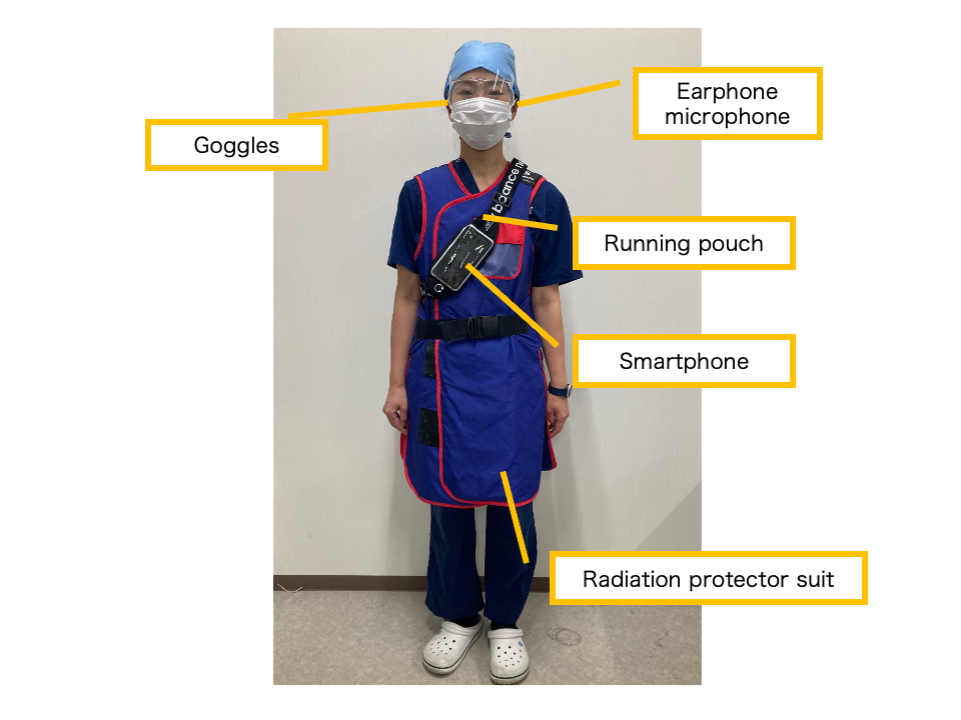
The equipment was prepared for nurses for voice activation.

Figure 2 illustrates the setup used for the voice input activation. The nurses wore standard protective gear, including radiation-proof clothing and goggles. A running pouch containing a smartphone and a wireless microphone was used for the voice input operation. The pouch was designed to fit securely onto the body, prevent interference with movement, and allow nurses to operate the device without removing it. As handwritten documentation remained in the official nursing record, the nurses also carried a traditional binder for handwritten entries.

### Training Procedures

Before the study, the participants registered their voice profiles on a smartphone to enhance their speech recognition accuracy. They activated and interacted with Google Assistant to confirm system responsiveness and learn the appropriate voice volume for reliable activation. The nurses were instructed that each interaction should record a single item per dialogue turn and were trained on how to verify the recorded content on the smartphone screen, if necessary.

### Data Collection Procedures

The target period for the voice input documentation ranged from patient entry to exit. To reduce workload during the study, vital signs and fluid balance data were excluded, allowing nurses to focus on narrating their procedural actions (what they did) rather than continuous physiological monitoring. The content to be recorded by voice input is assumed to correspond mainly to the “procedure progress” items shown in Figure 1.

For each case, the number of voice-input log entries was compared with the number of corresponding handwritten entries for the same phase. The ratio of voice input entries to handwritten events was calculated by setting the number of handwritten entries to 100. Each voice input log was cross-checked against the paper records to confirm that the events were successfully captured.

As the input accuracy of voice recognition systems has been partially validated in previous studies [19,20], this study did not reexamine transcription accuracy; instead, it focused on the feasibility and contextual barriers to real-time use.

### Observation Procedures

To gain a contextual understanding of the environment, real-time observation of nurses’ activities was used rather than video recordings. The research team observed nurses’ behavior in an operations control room adjacent to the catheterization laboratory, from the patient’s entry through to exit. An unstructured observation method, rather than a predefined checklist, was used. Observers recorded events and behaviors in free-text notes, focusing on how nurses interacted with the system and the contextual factors that influenced their use of voice input.

Each nurse’s voice input memos saved to Google Keep were shared with the observer’s account, allowing the observer to verify the timing of each voice input event in real time. The observers also noted the nurse’s location and concurrent actions at the time of recording.

### Usability Survey and Interviews

After each procedure, nurses participated in short face-to-face interviews regarding their experiences using the voice input system. Subsequently, a questionnaire using a 5-point Likert scale was distributed to collect structured feedback. The survey questions were developed with reference to established instruments such as the system usability scale. As this study aimed to identify barriers to system implementation in clinical settings, the questions were tailored to practical aspects and designed to facilitate participant response. Responses were collected online. Details of the items are provided in Multimedia Appendix 1: interview and questionnaire items.

### Ethical Considerations

This study focuses on patient safety and confidentiality. The study protocol was reviewed and approved by the Kyoto University Graduate School and Faculty of Medicine Ethics Committee (approval number R3508). Before participation, the purpose and procedures of the study were explained to all nurses, patients, and attending physicians, and their consent was obtained. The collected data were deidentified and strictly managed in a secure environment. No financial compensation was provided to the health care professionals.

## Results

### Overview of Cases and Participants

Between December 2022 and January 2023, 8 patients were studied in the catheterization laboratory. The participating nurses were 1 male and 4 females (4 aged in their 40s and 1 in their 50s). Throughout the sessions, the system configuration did not exhibit any connectivity or stability problems. In all cases, nurses provided handwritten documentation as an official record while attempting voice input whenever feasible.

### Ratio of Voice Input to Handwritten Records

Table 1 summarizes the case ID, assigned nurse, procedure duration, procedure type, number of voice input entries per case, number of handwritten entries on the record sheet, and percentage of voice input entries relative to handwritten entries.

In this pilot study, vital signs and fluid balance were intentionally excluded from voice input targets to reduce workload; target items spanned events from patient entry to exit (eg, admission or discharge times, catheter insertion, and administered medications). Although some procedures were lengthy, the number of recordable events was not necessarily proportional to the procedure time because longer durations often reflected extended physician operation times rather than increased nursing events.

**Table 1.** Experimental results.

Case	Nurse	Operating time (minutes)	Surgical procedure	Entries, n		VE/HW (%)
				VE^a^	HW^b^	
1	A	58	PTCD^c^ catheter replacement	8	16	50
2	A	110	PTCD catheter replacement, liver biopsy	9	19	47
3	A	59	PTCD catheter replacement	7	15	46
4	B	123	Transjugular vein liver biopsy	10	22	45
5	B	40	PTCD catheter replacement	7	18	38
6	C	174	PTCD catheter implantation	22	29	75
7	D	144	TACE^d^	16	25	64
8	E	199	PTCD catheter implantation	9	22	40

a VE: voice entry.

b HW: handwritten .

c PTCD: percutaneous transhepatic cholangiodrainage.

d TACE: transcatheter arterial chemoembolization.

### Phases Where Voice Input Was or Was Not Feasible

**Figure 3.**
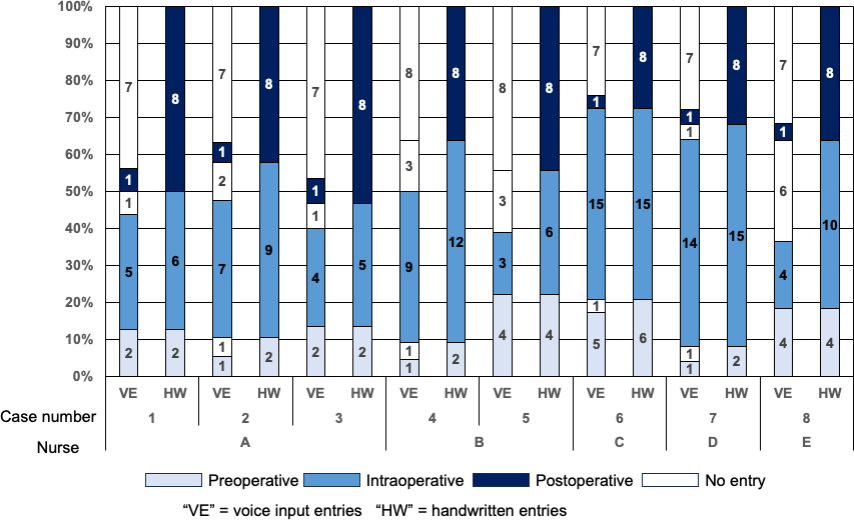
Ratio of voice input to handwritten input in each phase.

As shown in Figure 3, voice input captured 50% to 100% of the target items in the preoperative phase and 40% to 100% in the intraoperative phase, but only 0% to 12% in the postoperative phase.

Consistent with Figure 1, both the preoperative and postoperative phases involved dense task clustering within less than 10 minutes. While preoperative entries were often feasible by voice input, postoperative documentation was rarely recorded by voice, despite containing more items (eg, arterial palpation checks, puncture-site compression method, pressure-release timing, and verification of procedural steps). In one case, the procedure was temporarily paused because of the patient’s pain, and no voice input entry was created for this event.

### Observational Findings on Nurses’ Behavior

Across all cases, nurses sometimes used voice input while providing care, but they also frequently stepped away from the patient or physician during short intertask intervals to make voice input entries. As a result, the time stamp of voice entry does not always coincide with the actual care time. The nurses occasionally reviewed their smartphone entries when brief opportunities arose.

In one instance, when a patient reported pain during the procedure, the nurse administered analgesics and remained with the patient until stable; during this period, the medication name and dose were not recorded by voice input.

### System Usability Survey

The response rate was 100%. Nurse A participated on 2 separate days (cases 1 and 3) and completed surveys for both; because the second participation did not involve prior training, the second-day response was excluded to avoid confounding, leaving 5 analyzed responses (Multimedia Appendix 2: distribution of nurses’ responses to the poststudy Likert scale survey).

Wearing a single earbud did not interfere with the nursing tasks. However, nurses reported the need to speak aloud to activate the system, and several perceived the dialogue or processing speed to be insufficient for the clinical pace. The ratings for “difficulty inputting while working” and “confidence in voice input” were mixed. All participants agreed that technical support was required for practical use. The following interview comments further illustrate these tendencies: a button-based start might be preferable to voice wake-up; domain dictionaries could reduce recognition errors; with familiarity, hands-free narration (eg, reading out drainage amounts) might streamline tasks; recognition was often poor near electrocardiography monitors; a fixed smartphone placement might help; ear discomfort occurred with prolonged wear, suggesting other form factors for long procedures; speaking in a loud voice felt embarrassing; visible near-mouth devices (eg, smartwatch or handheld push-to-talk or intercom-style microphone) may be more acceptable; and potential use cases include night shifts or emergencies and intensive care unit or closed units.

### Integrated Findings From Logs and Surveys

Two patterns were consistent across cases: (1) postoperative items were rarely captured by voice input and (2) nurses often moved away from others to make voice input entries. The chosen earbud microphone was lightweight, unobtrusive, and beneficial for comfort; however, because it was not visibly apparent, others could not tell when voice input was being used. Combined with occasional activation failures (necessitating louder speech), nurses sometimes felt compelled to speak suddenly and loudly, which appeared socially awkward. This likely contributed to the tendency to step aside from the input.

Perceived system responsiveness is also considered suboptimal for busy workflows. Although brief prestudy training was provided, all participants indicated that additional technical support (eg, troubleshooting, supplementary training, and device optimization) would be required for sustained, routine use.

## Discussion

### Principal Findings

This pilot study examined the feasibility of hands-free and eyes-free voice input in real nursing workflows within a catheterization laboratory. Although the study focused on procedures with similar nursing tasks, the number of recorded voice input items varied by phase and content. These results suggest that the current voice dialogue system is not yet fully compatible with the realities of nursing workflows, and that nurses must constantly negotiate interactions with both the system and its surroundings. The following discussion considers the key barriers to the concurrent use of voice input and care tasks.

### Task Complexity and Cognitive Load

Despite the high workload in the preoperative phase, the documentation content—such as “patient entry,” “monitoring start,” and “procedure start”—was relatively standardized and consistent across patients. As sudden changes in the patient’s condition are uncommon, voice input is feasible for most items, resulting in a high input rate (50%-100%). During the intraoperative phase, voice input also achieved moderate to high rates (40%‐100%). This appeared to be related to the longer duration of this phase and fewer interactions between nurses, physicians, and patients, thereby providing brief moments suitable for voice-based recording.

In contrast, the postoperative phase showed extremely low input rates (0%‐12%). This period involves increased documentation and communication demands such as confirming postoperative instructions with physicians or explaining precautions to patients. Unexpected events, such as pain or unstable vital signs, often require immediate action. These conditions heighten nurses’ cognitive load and necessitate flexible prioritization, leaving a limited capacity for voice input. Similar findings were reported in the study by Chen et al [25], which noted that a greater task complexity reduces the feasibility of speech-based documentation.

In an environment where privacy was protected, nurses could perform voice input simultaneously with their tasks for simple and standardized content. However, the conflict between system interaction and clinical tasks and the increasing complexity of tasks have made their use difficult. Although further training and system customization may improve usability over time, situations in which voice input is inappropriate or technically unreliable remain. Therefore, backup methods such as quick manual notes or onscreen edits should be available to ensure the completeness of documentation.

### Context of Voice Input Use

Observations revealed that nurses often moved away from patients or physicians to provide voice input. This may reflect concerns about being interrupted or uncomfortable speaking aloud when others could not observe the interaction. Several nurses reported feeling embarrassed when they spoke loudly to activate the system. The inability of the system to recognize soft voices likely reinforces this behavior.

In one case, the nurse chose not to record the administration of analgesics while attending to a pain-experiencing patient. She expressed concern that saying aloud, “The patient is in pain and pain relief medication has been administered,” might make the patient anxious. These findings highlight that voice input is not merely a technical act; it is also embedded in the social and emotional contexts of care. In some situations, verbalizing information is inappropriate or psychologically uncomfortable for both nurses and patients.

Prior studies [18-20] have mainly evaluated voice input as a point-of-care aid in which nurses looked at a screen while dictating. This approach focuses on efficiency within conventional input workflows. In contrast, this study explored simultaneous voice input during care, which allows documentation without diverting gaze or hands, but may also shift the nature of documentation toward something shared and audible to the patient. This difference implies that introducing voice input for concurrent care requires consideration of the context, interpersonal dynamics, patient perceptions, and system usability.

Both environmental and social support are required to facilitate acceptance. In the initial stages, cooperation between health care staff and patients is essential. Creating an environment in which nurses can use voice input comfortably without fear of misunderstanding or interruption is a key step toward sustainable integration.

### Technical and Implementation Considerations

The findings suggest two primary barriers to real-time voice input in clinical settings: (1) the instability of relying solely on voice input as the main documentation method and (2) the gap between voice input and traditional input workflows. Therefore, several technical and environmental improvements are required to overcome these limitations. Microphones should detect lower-volume speech and ideally enable whisper-based recognition [26]. Custom wake words, faster response times, and the ability to enter multiple items in a single interaction could enhance efficiency. Additionally, context-sensitive “code words” or predefined phrases could allow nurses to record sensitive information discreetly when speaking near patients.

The microphone did not interfere with nursing tasks; however, it was found that having the device visible to others was preferable. Moreover, the potential physical effects of prolonged use should also be considered. The advantages and disadvantages of each device are listed in Table 2.

Making the input process more visible—for example, by using microphones attached to uniforms or earphones that illuminate when recording—could promote transparency and reduce discomfort. Smartwatches may also be practical for quick activation; however, some institutions restrict their use for hygiene reasons [27].

Nurses should communicate with patients and colleagues regarding voice input use. It is important for patients to understand that they can always talk to a nurse, even during the recording. Preoperative explanations can alleviate potential discomfort and encourage open communication. Voice input can also enhance safety during “*time-out* procedures [28], where audible confirmation of information benefits the entire surgical team. Beginning with such secure and cooperative contexts may help normalize technology.

**Table 2.** Advantages and disadvantages of each device.

Device type	Does not block the ears	Visibly worn	Compactness and lightness	Features
In-ear earphone- microphone	X	X	✔︎	The timing of input is difficult to share
Neck speaker	✔︎	✔︎	X	Sound is audible to the surrounding people
Open-ear type	✔︎	✔︎	✔︎	The ear-hook type may interfere with goggles
Headset (intercom type)	✔︎	✔︎	X	The ear-hook type may interfere with goggles
Smartwatch	✔︎	✔︎	✔︎	Wearing devices below the elbows is prohibited in some facilities

### Future Directions and Practical Implications

On the basis of the pilot findings, three potential directions for future development were considered: (1)

Regardless of the approach, improvements in the device design, targeted training, and collaborative understanding among health care staff can reduce the psychological and operational burdens of voice input. Beginning with environments and content that foster safety and comfort and gradually expanding their use may allow nurses to adapt to the system more naturally.

Overall, this pilot study provided preliminary insights into the barriers and contextual factors affecting voice input in nursing documentation. While these findings are not generalizable owing to the limited sample size and controlled setting, they highlight the essential design, training, and social considerations for future large-scale evaluations.

### Limitations

This study was conducted as a pilot investigation within a real clinical environment, with careful consideration of patient safety and minimization of the burden on participating nurses. Consequently, this study has several limitations that must be acknowledged.

First, the study was conducted in the catheterization laboratory of a single facility with a limited number of participants and a short study period. These constraints were intended to ensure clinical safety and avoid disrupting nursing workflows; however, they naturally limit the scope of interpretation.

Second, the input rate and nurses’ experiences with voice input may vary depending on the type of procedure, facility characteristics, and organizational culture used. Broader multisite investigations are needed to confirm whether the findings observed here are consistent in other contexts.

Third, as the study was conducted alongside routine clinical duties, the nurses were not solely dedicated to voice input. They continued their official handwritten documentation, which comprised their primary records. Therefore, voice input was used as a supplementary capacity. The results may differ if nurses rely exclusively on voice input without concurrent handwritten notes.

Additionally, this study did not assess changes over time, for example, how input rates or usability perceptions might evolve with continued use and increased familiarity. Therefore, the observed outcomes should be interpreted as preliminary findings reflecting the early stages of the system introduction.

Despite these limitations, this pilot study offers valuable preliminary insights into the contextual and technical barriers to implementing hands-free and eyes-free voice inputs in real-world nursing settings. These findings provide practical guidance for future research on system refinement, workflow integration, and user adaptation under varying workload levels and interactions with other health care professionals.

### Conclusions

This pilot study explored the feasibility of hands-free and eyes-free voice input in clinical settings within a catheterization laboratory. These results suggest that nurses were able to use voice input concurrently with their tasks in situations that imposed relatively low cognitive demands. Conducting the study in an actual clinical environment also revealed psychological factors such as tension, hesitation, and environmental constraints, which cannot be replicated in simulations.

In this preliminary stage, two major barriers to introducing voice input in clinical workflows were identified: (1) the instability of relying solely on voice input as the main documentation method and (2) the gap between new voice-based methods and conventional input practices.

To reduce nurses’ cognitive and psychological burden, it is important to start with simple, low-risk documentation tasks and content that can be safely shared with other staff members or patients. Continued technical improvements, such as the recognition of softer speech and visual cues that indicate when the system is active, may further enhance its usability and acceptance.

Although limited in scope and duration, this pilot study provides preliminary insights into the contextual, technical, and human factors affecting the introduction of voice input in clinical practice. These findings serve as a foundation for future research aimed at developing safer, more adaptive, and context-sensitive systems for nursing documentation.

## Supplementary material

10.2196/71462 Multimedia Appendix 1 Poststudy survey for nurses.

10.2196/71462 Multimedia Appendix 2 Distribution of nurses’ responses to the postexperiment Likert scale survey.

## Abbreviations

PTCD: percutaneous transhepatic cholangiodrainage
TACE: transcatheter arterial chemoembolization
