# Supplementary figures and images for: Clinical Barriers to Hands-Free, Eyes-Free Voice Input for Nursing Records: Field Usability Study
Source: Asian Pac Isl Nurs J. 2026 Jun 2;10:e71462. doi: 10.2196/71462 (PMC13229460; doi:10.2196/71462)

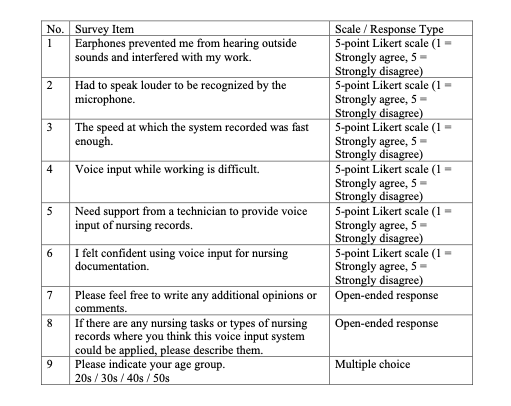

Supplement: Multimedia Appendix 1 [file apinj-v10-e71462-s001.png]

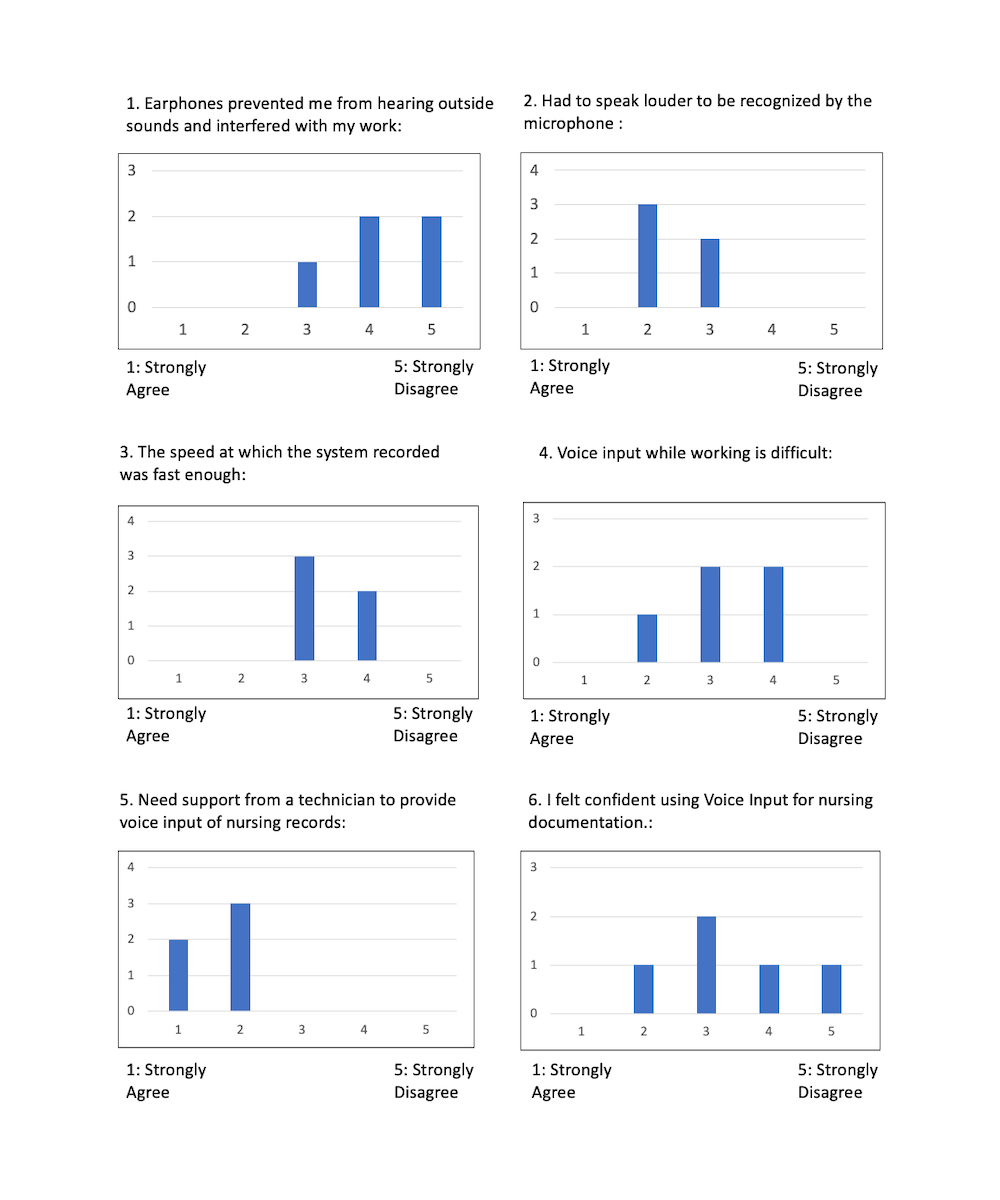

Supplement: Multimedia Appendix 2 [file apinj-v10-e71462-s002.png]
